# Evaluation of sealing ability and adaptability of different endodontic sealers: *in vitro* comparative study

**DOI:** 10.7717/peerj.21621

**Published:** 2026-08-04

**Authors:** Abdelrahman M. Alhilou, Abdulaziz Bakhsh, Reem Alosaimi, Maysan Garoot, Ayat Alshanqiti, Doha Jaha, Shahinaz Sembawa, Nadia Badr

**Affiliations:** 1 Department of Restorative Dentistry, Faculty of Dental Medicine, Umm Al-Qura University, Makkah, Saudi Arabia; 2 Faculty of Dental Medicine, Umm Al-Qura University, Makkah, Saudi Arabia; 3 Division of Dental Public Health, Department of Preventive Dentistry, Faculty of Dental Medicine, Umm Al-Qura University, Makkah, Saudi Arabia; 4 Department of Dental Biomaterials, October 6 University, Cairo, Egypt

**Keywords:** Endodontics, Root canal sealers, Scanning electron microscope, Tricalcium silicate

## Abstract

**Background:**

Root canal sealers play a critical role in preventing microleakage and ensuring the long-term success of endodontic treatment. AH-Plus is widely considered the gold standard epoxy resin–based sealer, while Bio-C sealer represents a newer premixed bioceramic material with bioactive properties. NanoSeal-S is a recently introduced silicone-based sealer reinforced with nanosilver particles, for which limited independent scientific data are currently available. Therefore, this study aimed to evaluate and compare the sealing ability and adaptability of these three root canal sealers.

**Materials and Methods:**

Forty-five extracted single-rooted human teeth were decoronated and randomly divided into three experimental groups (*n* = 15) based on the sealer used: Group A—AH-Plus, Group B—NanoSeal-S, and Group C—Bio-C sealer. Root canals were instrumented using the ProTaper Next system and obturated with the single-cone technique. Adaptability was assessed by calculating the percentage of sealer–dentin interfacial contact using stereomicroscopy and ImageJ analysis. Microleakage was evaluated using the dye penetration clearing technique. Viscosity of each sealer was measured using a Brookfield viscometer. Data normality was assessed using the Shapiro–Wilk test. Interfacial contact percentage data were analyzed using one-way ANOVA followed by Tukey’s *post-hoc* test, whereas microleakage data were analyzed using the Kruskal–Wallis test followed by Dunn’s multiple comparison test at a significance level of 0.05.

**Results:**

NanoSeal-S showed the highest interfacial contact percentage (92.3%), followed by AH-Plus (75.8%) and Bio-C sealer (59.9%), with a statistically significant difference (*P* < 0.001). Bio-C sealer exhibited the lowest median microleakage value (0 µm), whereas NanoSeal-S demonstrated the highest leakage (166 µm). AH-Plus showed intermediate leakage values (65 µm). Viscosity measurements showed NanoSeal-S had the lowest viscosity, followed by AH-Plus and Bio-C sealer.

**Conclusion:**

NanoSeal-S demonstrated superior interfacial adaptability, whereas Bio-C sealer showed the least microleakage among the tested materials.

## Introduction

The success of endodontic treatment is achieved through the eradication of microorganisms from the dentin-pulp complex and the prevention of subsequent reinfection in the canal space or periapical area. This is mainly accomplished through chemo-mechanical preparation, followed by three-dimensional filling of the root canal system with an inert biocompatible material (Nawal et al., 2011). Because of their ability to hermetically seal the canal space, endodontic sealers play a critical role as obturating materials. The sealers should be non-irritating to oral tissues, non-staining to hard dental tissues, dimensionally stable, radiopaque, insoluble in tissue fluids, and should not encourage bacterial growth (Hergt et al., 2015). By adhering to dentin, sealers should prevent any leakage between the obturating material and the dentin wall, as well as avoid dislodgement of the root canal filling (Rashid, 2021).

The physiochemical properties of root canal sealers are important factors in endodontic treatment because they affect the quality of filling material (Ørstavik, 2005; Ørstavik, Eriksen & Beyer-Olsen, 1983). These properties, such as viscosity, flowability, film thickness, and solubility, are based on the constituents of the material used. The flowability of an endodontic sealer determines its extent of engagement into minute spaces, especially in anatomically complex root canal systems. Sealers should have low viscosity to flow and penetrate the dentinal tubules for efficient adaptability, which minimizes the interfacial gap between the sealer material and the radicular dentin, in addition to preventing microleakage (Arikatla et al., 2018; Zhou et al., 2013; Şen, Pişkin & Baran, 1996).

Microleakage at the sealer–dentin interface remains one of the main factors that may compromise the long-term success of endodontic treatment. Even when the canal is adequately shaped and obturated, incomplete adaptation of the sealer to dentinal walls may create interfacial gaps that permit the passage of fluids, bacterial by-products, or microorganisms (Muliyar et al., 2014). The quality of this interface is influenced by the physicochemical behavior of the sealer, particularly its viscosity, flowability, dimensional stability, and ability to maintain intimate contact with root canal dentin (Keskin et al., 2026). A sealer with appropriate flow and low film thickness can better penetrate irregularities, lateral canals, and dentinal tubules, thereby improving adaptation and reducing void formation. However, excessive flow or inadequate dimensional stability may negatively affect the thickness and continuity of the sealing layer (Juntha et al., 2024). Therefore, evaluating the interfacial adaptation and microleakage resistance of different sealers is essential to determine their potential clinical performance.

Microleakage is “the diffusion of the bacteria, oral fluids, ions and molecules into the tooth and the filling material interface” (Muliyar et al., 2014). Microleakage may occur apically through different pathways such as root canal filling techniques, chemical and physical properties of the root canal filling materials, and the presence or absence of the smear layer.

Endodontic sealers have been continuously developed and modified over several years, with the goal of achieving an optimal sealer with enhanced physicochemical properties that facilitates successful obturation, thereby preventing the leakage of contaminants such as saliva, bacteria, and periapical tissue fluid into the canal space (Nawal et al., 2011). The use of nanotechnology and bioceramic osteo-promotion has been claimed to improve the physicochemical properties of root canal sealers (Ahmed, Ghani & Mahmoud, 2025).

A wide range of sealers are currently available and are classified according to their biological and physical properties as well as their chemical composition. Epoxy resin-based sealers have traditionally been regarded as the gold standard due to their favorable characteristics. Recent advancements have focused on developing sealers utilizing newer technologies to improve endodontic obturation and increase the success rate of endodontic therapy (Błaszczyk-Pośpiech et al., 2025).

One such sealer is NanoSeal-S, a silicone-based root canal sealer containing nanosilver for its antibacterial properties, supplied as a two-paste system. It is eugenol-free, radiopaque, and slightly expands upon setting for improved seal (Table 1). Studies confirm uniform micro-silver and about 0.2% expansion for improved adaptation (Verma, Arora & Taneja, 2024). Ingredients include siloxanes, paraffin oil, and platinum catalyst, in line with VPS chemistry (Verma, Arora & Taneja, 2024). While NanoSeal-S’s high flow and slight expansion help sealing, some research has found lower bond strength and penetration *vs*. other sealers, so further testing is needed (Verma, Arora & Taneja, 2024; Madhuri et al., 2016). Another widely referenced resin-based sealer is AH-Plus (Dentsply Maillefer, Ballaigues, Switzerland), which has achieved gold standard status in large part due to its consistent sealing ability as documented in the literature. This product is formulated as a two-component epoxy resin system (paste-paste) and includes a range of constituents, notably bisphenol-A, bisphenol-F, calcium tungstate, zirconium oxide, aerosil, and iron oxide pigments. Epoxy resin sealers, such as AH-Plus, are commonly used as benchmarks in comparative research. They generally offer low solubility and stable physical characteristics (Almeida et al., 2020; Zhang, Li & Peng, 2010; Eldeniz et al., 2007).

**Table 1:** Types, composition, and manufacturers of used sealers.

Sealer	Type	Composition	Manufacturer
AH-Plus	Epoxy resin-based	Paste A: bisphenol—an epoxy resin, pisphenol-F epoxy resin, calcium tungstate, zirconium oxide, iron oxide pigments, silica. Paste B: dibenzyloxanonadiamine, adamantaneamine, tricyclodecanediamine, calcium tungstate, zirconium oxide, silica, silicon oil.	Dentsply Sirona, Germany
NanoSeal-S	Silicon-based	Polydimethylsiloxane, nanosilver, silicon oil, platinum catalyst, nano zirconium dioxide, paraffin liquid (excipient).	Prevest Den Pro, India
BIO-C	Calcium silicate	Calcium silicates, calcium aluminate, calcium oxide, zirconium oxide, iron oxide (pigmentation), silicon dioxide (rheology agent), and polyethylene glycol (dispersing agent).	Angelus, Brazil

Bio-C Sealer (Angelus, Londrina, PR, Brazil) is a calcium silicate-based material noted for its favorable flow, thin film thickness, and alkaline pH, which enhances its antibacterial properties. It also sets quickly and is designed to ensure an effective seal. Sealers like Bio-C are known for their alkaline environment and calcium-ion release, features linked to bioactivity (Silva et al., 2020). However, a study showed that some of these materials may have higher solubility than ISO standards allow, which could impact their long-term sealing ability depending on how they are tested and stored (Zordan-Bronzel et al., 2019).

Therefore, the aim of this study is to evaluate the interfacial adaptation of different sealers. This study assesses NanoSeal-S and Bio-C sealers to the dentinal wall of endodontically treated teeth and evaluates their sealing ability in preventing microleakage. The obtained results of these two sealers are then compared with those of AH-Plus. The null hypothesis is that there is no significant difference between different sealers regarding adaptability and sealing ability.

## Materials and Methods

Three commercially available endodontic sealers (NanoSeal-S, Bio-C, and AH-Plus) were used in this study to compare their sealing ability and adaptability. The investigated endodontic sealers in this study are shown in Table 1 with their compositions.

### Ethical approval

The protocol of this study was approved by The Biomedical Research Ethics Committee at Umm Al-Qura University (Approval no. HAPO-02-K-012-2023-02-1457), and informed written consent was obtained from all patients prior to tooth extraction.

### Selection of sample

Forty-five permanent single-rooted human teeth with fully developed root apices, extracted for unknown reasons were collected from Umm Al-Qura university dental clinics. Consent was taken from patients to collect these teeth for research purposes. All teeth with canal calcifications, immature apices, multiple canals, root fracture, and root resorption were excluded. The sample size calculation was done with 80% power and 5% alpha using an online sample size calculator (https://clincalc.com/stats/samplesize.aspx) and was determined based on a previous study (Trivedi et al., 2020).

### Preparation of sample

**Figure 1:**
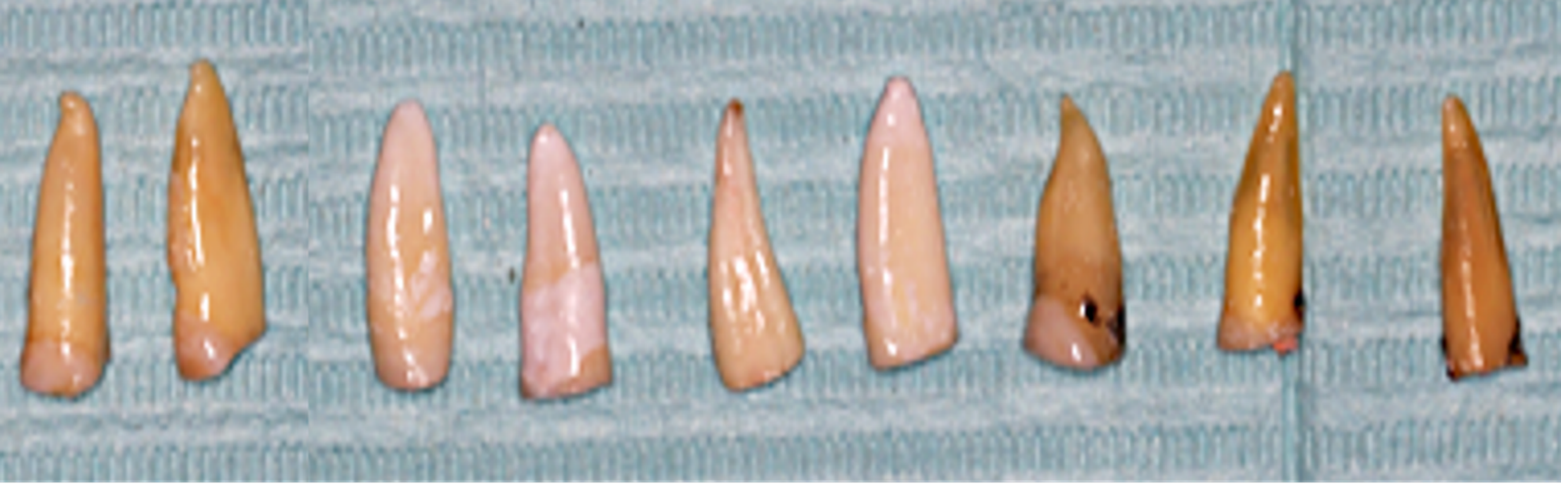
Samples used for one of the assigned groups.

The teeth were washed under running water to remove external debris and stored in 0.9% normal saline at 37 °C. The selected samples were decoronated using a diamond disc away from the apex by an average of 16 mm. These samples were then randomly distributed into three groups, with each group containing 15 teeth (Fig. 1): *Group A*: AH-Plus sealer (epoxy resin-based), *Group B*: NanoSeal-S sealer (rubber-based), and *Group C*: Bio-C sealer (tricalcium silicate-based). Pulp was extirpated and a #15 K-file was inserted into the canal until it was visible at the apical foramen. Working length was determined after subtracting 1.0 mm from the obtained measurement.

### Root canal preparation

A glide path was created using K-files, ending with the #25 K-file (MANI, Tochigi, Japan). All root canals were prepared with the step-back technique using a rotary X-Smart endodontic motor (Dentsply Sirona, Charlotte, NC, USA) with ProTaper Next X1, X2, and X3 rotary files (Dentsply Sirona, Charlotte, NC, USA). In between each file, canals were irrigated with 10 mL of 5.25% sodium hypochlorite (Diaa Dental Products, Riyadh, Saudi Arabia). Final irrigation was performed with 3 mL of 17% Ethylenediaminetetraacetic acid (EDTA) solution (Diaa Dental Products, Riyadh, Saudi Arabia) to remove the smear layer and the canals were dried using paper points. Finally, the master cone was placed inside the canals and checked radiographically.

### Obturation

**Figure 2:**
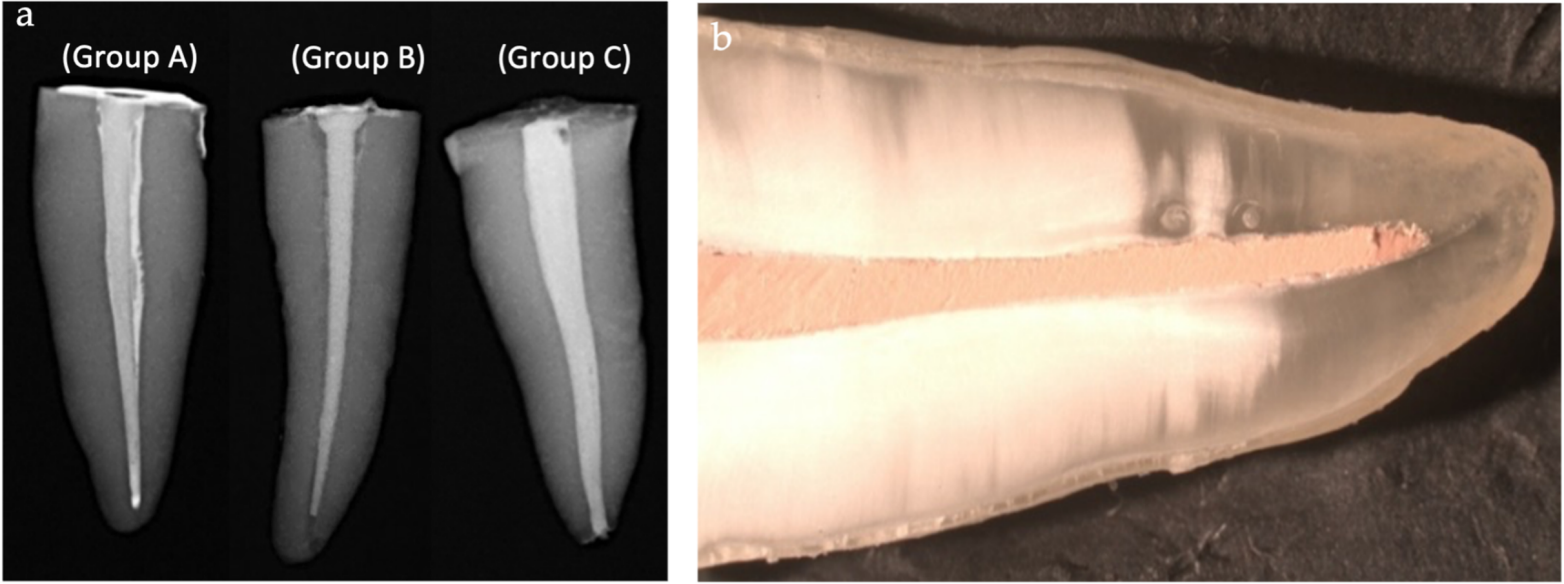
(A) Radiographs of the obturated samples; (B) a stereomicroscope image of a sectioned root (10×).

All three groups were obturated using the single-cone technique. A ProTaper Next Gutta-Percha (X3) was coated with one of the investigated sealers and inserted into the canal with cones seared off using a heated plugger. Radiographs were taken to assess the quality of the root canal filling (Fig. 2A). Later, the external surfaces of the decoronated teeth were completely coated with two layers of nail varnish except for 1 mm at the apex, which remained exposed. All samples were stored for 7 days at 37 °C in 100% relative humidity in sealed containers containing moistened gauze to ensure complete setting of the sealers under simulated intraoral conditions.

### Assessment of adaptability

After the sealers were fully set, five roots of each group were longitudinally sectioned, giving ten samples per group, using a rotary microtome MY T63C2 (IEC 34-1, IEC 34-5 (1983); Tecnomotori, Collecchio, Italy) to measure the interfacial gap between the sealer and the radicular dentin. The ten sectioned root samples were then analyzed under stereomicroscope (CH-9435 Heerbrugg with Leica DFC 290 Camera; Leica Microsystem, Wetzlar, Germany) at 10× magnification, which allowed visualization of the entire sealer–dentin interface for standardized gap assessment.

### Interfacial contact analysis

**Figure 3:**
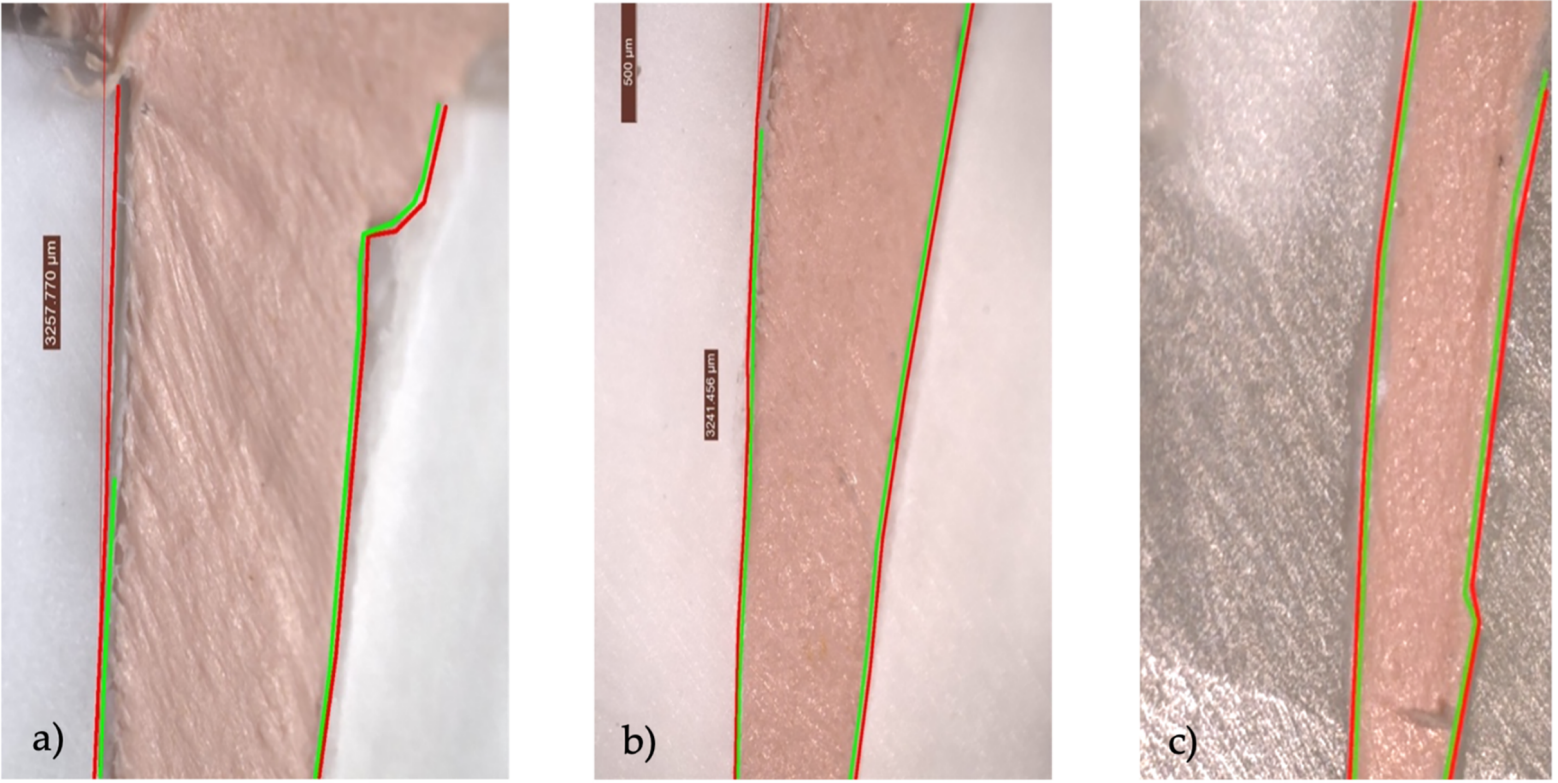
Quantification of sealer–radicular dentin interfacial contact. The entire visible sealer–dentin interface of each section was traced and measured (red line), and the portions showing direct contact between the sealer and radicular dentin were measured separately (green line). The percentage of contact was calculated according to the displayed equation. Representative images are shown from the: (A) coronal third, (B) middle third, and (C) apical third of the root.

The interfacial contact of the sealers to the dentinal wall was measured for adaptation assessment. Each root was divided horizontally into three segments: coronal, middle, and apical parts. The stereomicroscope images of the sectioned obturated half roots (Fig. 2B) were analyzed using the computer software program ImageJ version 1.54d. This software was run on a computer with a Microsoft Windows 11 Pro (64-bit) operating system. For the calculation of sealer/radicular dentin interfacial contact percentage, the whole length (in µm) of the radicular dentin at the interface was measured by the “Segmented Line” tool in the software, and the length was recorded (red line). Then, the lengths (in µm) of sealer in contact only with the radicular dentin at the interface were calculated by the segmented line tool (green line); Fig. 3. The sum of the green segments, representing the entire length of the sealer in contact with the whole length of the radicular dentin (represented by the red line), was used to calculate the percentage of contact according to the following equation:

$Percentage\; of\; contact = \displaystyle{{Sum\; of\; green\; lines\; lengths} \over {Entire\; length\; of\; red\; line\; }}\; \times 100.$

### Microleakage analysis

The remaining 10 decoronated teeth in each group were separately immersed in Indian ink (Atom Scientific, Rexcine Way, United Kingdom) and stored at 37 °C for 1 week. The teeth were then washed, and the varnish was removed with acetone. Samples were decalcified in 5% nitric acid (Sigma-Aldrich, Burlington, MA, USA) for 5 days; the acid was changed daily and agitated several times a day. After decalcification, the teeth were washed in water and then dehydrated successively in ascending grades of ethanol starting with 70% overnight, then 80% for 4 h, 90% for 2 h, and 100% for 1 h. Finally, the teeth were placed in methyl salicylate (Sigma-Aldrich, Burlington, MA, USA) for 60–90 min to render the samples transparent (clearing technique) (Khan, Rehman & Habib, 2015).

After decalcification and clearing, dye penetration was measured linearly (in µm) from the apical foramen to the maximum extent of dye penetration using calibrated ImageJ software (Version 1.54d; National Institutes of Health, Bethesda, MD, USA). Calibration was performed using a stage micrometer prior to measurements.

**Figure 4:**
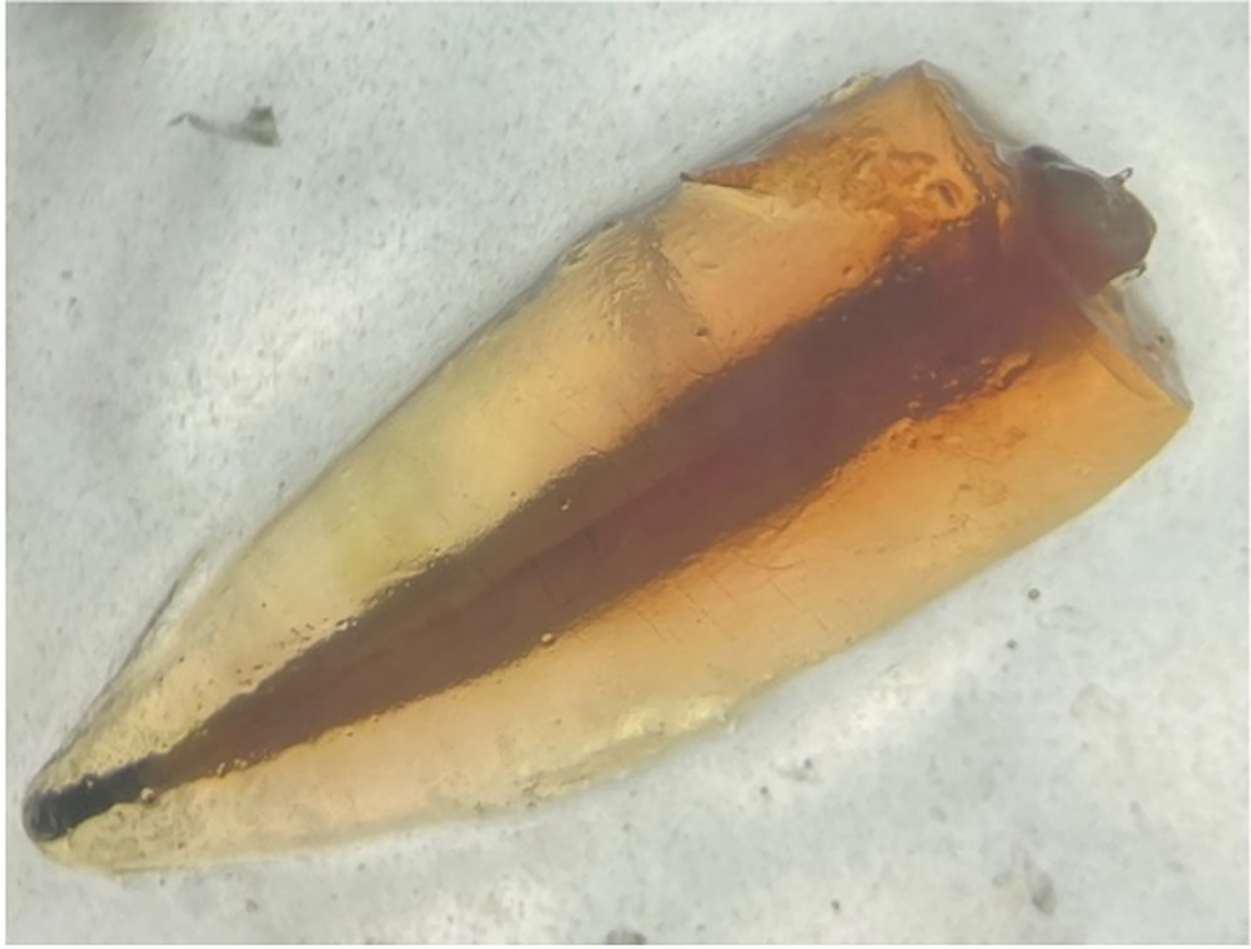
A cleared sample for microleakage assessment of AH-plus sealer.

Two independent blinded examiners performed the measurements. Inter-examiner reliability was assessed using intraclass correlation coefficient (ICC = 0.91), indicating excellent agreement. The mean of the two readings was used for statistical analysis (Fig. 4).

### Viscosity test

The viscosity of the selected sealers was measured using a viscometer (Brookfield DV2 Viscometer, Middleboro, MA, USA). One mL of each sealer was dispensed on a glass slab and tested under a torque range of 70% to 85%, a speed of 20 RPM, and at a temperature of 21 ± 1 °C for 2 min. Three measurements were recorded for each material, and the mean viscosity value (centipoise, cP) ± standard deviation was calculated and compared with those of the other sealers.

### Scanning electron microscope (SEM) analysis

A representative sample for each group was selected for scanning using a scanning electron microscope (JEOL-JSM 7600 FEG; FE-SEM, Peabody, MA, USA) at a 150× magnification to examine the adaptability of the sealer at the sealer/radicular dentine interface. The selected specimens were mounted on an aluminum stub, vacuum dried, and sputter coated with gold to thoroughly examine the surface morphology.

### Statistical analysis

Statistical analysis was performed using SPSS version 28 (IBM Corp., Chicago, IL, USA). Significance level was set at α = 0.05. Data normality was assessed using the Shapiro–Wilk test. Interfacial contact percentage data were normally distributed and analyzed using one-way ANOVA followed by Tukey’s *post-hoc* test. Microleakage values were non-normally distributed and analyzed using the Kruskal–Wallis test followed by Dunn’s multiple comparison test.

## Results

The mean and standard deviation of interfacial contact (IF) and the corresponding contact percentage of sealers/radicular dentin as well as the viscosity of the sealers are presented in Table 2. A statistically significant difference was evident among the three endodontic sealers (*P* < 0.001). NanoSeal-S recorded the highest significant contact percentage value (92.3 ± 0.05) while Bio-C recorded the lowest significant contact percentage value (59.9 ± 0.04). AH-Plus recorded an intermediate contact percentage (75.8 ± 0.03).

**Table 2:** The means and standard deviation of interfacial contact (IF) in mm and the corresponding contact %. The viscosity in cP.

Sealer type	Contact % (mean ± SD)	Viscosity (cP)
AH plus	75.8 ± 0.03^b^	238.8
NanoSeal-S	92.3 ± 0.05^a^	210.0
Bio-C sealer	59.9 ± 0.04^c^	255.3

**Note:**

Values are presented as mean ± standard deviation. Overall group comparisons were performed using one-way ANOVA followed by Tukey's *post hoc* test. Different superscript letters (a–c) indicate statistically significant differences between groups (*P* < 0.05).

**Figure 5:**
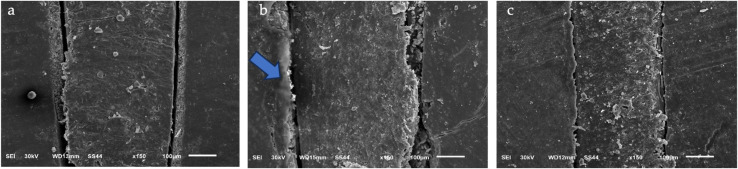
SEMicrographs of obturated root canals with different sealers at magnification ×150. (A) AH plus micro-gaps are present on both sides of the 2D-image either at the sealer/radicular dentin interface or the sealer/GP interface. (B) Nano seal-S micro-gap at the left side (pointed by the arrow) exhibits the rubbery sealer which overlaps the gap. Meanwhile, the other side shows remnants of the sealer interrupted in the micro-gap which might be torn during the obturation procedure. (C) Bio C sealers the 2D-image of this sealer shows the least interfacial gap among the investigated sealer. White spots denote ceramic particles.

When investigating the median leakage among all three types of sealers, Bio-C recorded a median leakage value of 0 µm, indicating leakage below the detection threshold of the dye penetration method used in this study. This value was significantly lower than that of NanoSeal-S (166.44 µm; *P* < 0.02). AH-Plus recorded a lower leakage value (65.92 µm) than NanoSeal-S, with no statistically significant difference when compared to the other sealers (Table 3 and Fig. 5).

**Table 3:** The median of dye penetration in micrometer. Bio-C had a median leakage of zero micrometer (µm), which was significantly different from nano seal and AH plus sealers.

Sealer type	*N*	Median (IQR)	*P*-value
**AH plus**	21	65.92 (0.00–258.06)	
**Nano seal-S**	20	166.44 (0.00–438.89)	<0.001
**Bio-C sealer**	19	0 (0.00–33.71)	

## Discussion

None of the investigated sealers could thoroughly coat the gutta-percha and the walls of the canal, nor fill the spaces. The close adaptation of gutta-percha to the dentin wall would squeeze the sealer and interrupt its continuity, forming voids between the gutta-percha and dentin interface or displacing the compressed gutta-percha (Setya et al., 2014). The present study confirmed this fact as no 100% contact percentage was obtained with any of the investigated sealers.

It should be noted that the zero-leakage value recorded for Bio-C should not be interpreted as a complete absence of leakage. Rather, it indicates that leakage, if present, was below the detection threshold of the dye penetration methodology employed in this study. As with other leakage assessment techniques, dye penetration has inherent limitations and may not detect very small leakage pathways. Therefore, the findings should be interpreted as a comparative evaluation of sealing performance among the tested materials rather than evidence of complete sealing ability.

The interfacial contact percentages observed between the sealers and radicular dentin denoted the presence of significant differences among the investigated sealers, where NanoSeal-S recorded the highest contact percentage (92.3 ± 0.05), followed by AH-Plus (75.8 ± 0.03), while Bio-C exhibited the lowest contact percentage (59.9 ± 0.04). These results aligned with the measured viscosity results. As a basic physical concept, the more viscous the fluid is, the less tendency it has to flow. The high contact percentage recorded by NanoSeal-S could be attributed to it having the lowest measured viscosity. It also demonstrated thixotropic behavior as the viscosity decreased, with increased pressure allowing the sealer to flow more during the obturation technique (Dasari et al., 2020). NanoSeal-S also expanded very little (about 0.2%) upon setting, which also improved its contact percentage. Although the viscosity measurements were performed as a confirmatory test, the obtained results support the manufacturer claims that the flow character of each sealer complies with ADA Specification #57 and ISO 6876 Specification for root canal sealer flow (Zhou et al., 2013).

Al-Haddad, Kasim & Aziz (2015) found that bioceramic-based sealers exhibit more gap-containing regions and less interfacial adaptation when compared to AH-Plus. Their results align with the finding in the current study that showed the Bio-C sealer had the lowest interfacial contact percentage. Caceres et al. (2021) found that Bio-C had greater tubular adaptation than AH-Plus in all of the root canal.

Microleakage is defined as the clinically undetectable passage of bacteria, fluids, molecules, or ions between the tooth and the restorative material. It is one of the most common reasons for failure in endodontic treatment (Nawal et al., 2011). Microleakage may occur because of root canal filling techniques, the chemical and physical properties of the root canal filling materials, and the presence or absence of the smear layer. The composition and characteristics of the sealer are crucial to determining the adaptability of the sealer to radicular dentins and consequently, the sealer’s sealing ability (Tuncer & Tuncer, 2012). However, chemical composition is not the only determining factor of the flow characteristics and consistency of the sealer; setting reaction, solubility, and the adherence of sealers to the root canal wall are also influential (Gandolfi, Siboni & Prati, 2016; Asawaworarit, Pinyosopon & Kijsamanmith, 2020).

Sealers play an essential role in the success of root canal treatments. The area occupied by the sealer represents the weakest zone in the system due to setting changes and exposure to internal and external factors that may dissociate the material with time (Al-Haddad, Kasim & Aziz, 2015; Li et al., 2014). Microleakage has been attributed to the lack of an effective bond at the sealer/dentin and sealer/gutta-percha interfaces. Several trials have evaluated the sealing ability of root canal sealers by assessing the extent of microleakage (Hovland & Dumsha, 1985; Wu & Wesselink, 1993). However, these studies have significant disparities, and the results of these studies have been conflicting (Ferreira, Bombana & Sayeg, 2008).

An adequate entanglement between the radicular dentin and the sealer is essential for an evaluation of the adaptability of endodontic sealers (Caceres et al., 2021). Arikatla et al. (2018) postulated that minimizing the interfacial gap between the sealer material and the root canal wall would prevent microleakage. The results of the SEM analysis in the current study showed that none of the investigated groups was gap-free. However, the least significant recorded microleakage value was recorded by the Bio-C sealer, which showed the narrowest interfacial gap. This finding is consistent with the findings of previous studies. Srikumar et al. (2023) found that bioceramic sealers have the lowest dye penetration leakage, while other studies have demonstrated that NanoSeal-S has the highest dye penetration, which indicates the highest microleakage (Rashid, 2021; Saumya & Sumita, 2022).

Bio-C recorded the lowest microleakage, which may be because of the bioactivity of bioceramic sealers. Bioceramic-based sealers use the moisture naturally present in the dentinal tubules to commence and complete their setting reaction because the bioceramic-based sealers are soluble and hydrophilic (de Miranda Candeiro et al., 2012). The sealers deeply penetrate the dentinal tubule and form tags providing mechanical interlocking linkages that increase the retention of the root canal sealer (Antunovic et al., 2021). Moreover, hydroxyapatite crystals bond chemically to the root dentin wall along the infiltration zone, constituting mineral plugs that can obliterate the micro-space, creating a possible portal of entry for microorganisms (Pawar, Pujar & Makandar, 2014). This possibility was confirmed by SEM examination in the present study.

AH-Plus exhibited intermediate microleakage values compared with the other tested sealers, which corresponded to its intermediate contact percentage (75.8%) and viscosity (238.8 cP). This behavior may be related to the chemical composition, physical properties, and setting reaction of AH-Plus. The relatively large particle size (1.5–8 µm) may limit penetration into dentinal tubules, thereby reducing micromechanical interlocking. In addition, the polymerization process of epoxy resin-based sealers may contribute to the formation of interfacial gaps during setting. These factors may influence dye penetration and the overall sealing performance of the material (Camilleri, 2014). The SEM results support this explanation.

The apparent contradiction between the high interfacial contact percentage and the high microleakage observed for NanoSeal-S suggests that initial adaptation alone may not fully predict sealing performance. Although the thixotropic nature and low viscosity of NanoSeal-S may facilitate flow and intimate contact with the canal walls during obturation, the sealing ability of a material also depends on its behavior after setting (Desouky, Negm & Ali, 2019). Post-setting dimensional changes, stress relaxation, or localized separation at the sealer–dentin or sealer–gutta-percha interfaces may create pathways for fluid penetration despite the presence of extensive initial contact. In addition, surface tension and wettability play important roles in sealing performance (Ørstavik, Nordahl & Tibballs, 2001). A material with insufficient wettability may exhibit a high apparent contact percentage while still failing to establish intimate microscopic adaptation to the substrate. This may result in the formation of micro-spaces at the interface that are not readily detected during contact analysis but can permit dye penetration and contribute to microleakage (Bourgi et al., 2024). Therefore, interfacial contact and sealing ability should be considered complementary rather than interchangeable measures of sealer performance.

Surprisingly, the NanoSeal-S sealer recorded the highest contact percentage at the sealer/radicular dentin interface (92.3%) and had the least measured viscosity (210 cP) but recorded the highest measured microleakage. The main component of the NanoSeal-S sealer is polydimethylsiloxane. Polydimethylsiloxane belongs to a group of polymeric organo-silicon compounds that are commonly referred to as silicones. This silicon-based rubber sealer has unusual rheological properties, which are represented by thixotropic behavior. Its rubber nature might explain NanoSeal-S’s lack of sealing ability, due to its stiffer consistency and prominent surface tension, resulting in insufficient wettability (Zhou et al., 2013). The thixotropic behavior of this sealer allows it to flow under the pressure of obturation, leaving micro-spaces between the GP cone and the dentin wall where the sealer may pull away from the gutta-percha on setting and/or not bond completely with the radicular dentin (Tyagi, Mishra & Tyagi, 2013). SEM examination results support this explanation. It should be mentioned that there are contradictions regarding the sealing ability of silicone-based sealers; some studies have shown that these sealers have the top sealing performance (Gençoglu, Türkmen & Ahiskali, 2003; Ebert et al., 2014), while others identified them as having the worst (Barbizam et al., 2007; Sauod & Orafi, 2022).

The discrepancies among these studies may be attributed to differences in experimental design and evaluation methodologies. Variations in obturation techniques, root canal anatomy, sample preparation protocols, storage conditions, and setting times may influence the behavior of sealers and their sealing performance (Gharechahi et al., 2026). Furthermore, different microleakage assessment methods, such as dye penetration, fluid filtration, bacterial leakage models, and micro-computed tomography, evaluate different aspects of sealing ability and may produce divergent results. Differences in observation periods may also contribute, as some materials undergo dimensional changes, dissolution, or interfacial degradation over time. Therefore, caution should be exercised when comparing the results of different studies directly.

Several physicochemical properties influence the sealing ability of endodontic sealers, including viscosity, solubility, dimensional stability, polymerization shrinkage, and adhesion to dentin. In this study, viscosity was evaluated because it directly affects the flowability of the sealer and its ability to penetrate irregularities and dentinal tubules, which may influence interfacial adaptation. However, it should be noted that viscosity represents only one factor among many that contribute to the overall sealing performance of root canal sealers (Błaszczyk-Pośpiech et al., 2025).

It should be acknowledged that dye penetration methodology has inherent limitations. The molecular size of dye particles differs from bacterial dimensions, potentially resulting in over- or underestimation of leakage. Additionally, dye penetration lacks full standardization and may involve subjective interpretation. Although widely used and methodologically simple, dye-based microleakage assessment may yield results that differ from more objective techniques such as fluid filtration, bacterial leakage models, dye extraction methods, or micro-computed tomography. Moreover, the SEM evaluation in this study was performed at a relatively low magnification (150×), which provided a general overview of the sealer–dentin interface but may not allow detailed visualization of micro-gaps or sealer tags within dentinal tubules. Future studies using higher magnifications (*e.g*., 500–2,000×) may provide more precise characterization of the interfacial adaptation. Additionally, the batch numbers of the evaluated sealers were not recorded during specimen preparation, which limits complete material traceability.

Future studies should incorporate complementary methodologies, such as fluid filtration, bacterial leakage models, dye extraction techniques, and micro-computed tomography, together with higher-resolution SEM imaging and larger sample sizes, to provide a more comprehensive evaluation of the sealing ability and interfacial adaptation of root canal sealers. Therefore, the findings of this study should be interpreted within the limitations of the methodology.

## Conclusions

The null hypothesis was rejected, as significant differences were observed among the investigated sealers regarding sealing ability and adaptability. NanoSeal-S demonstrated the greatest interfacial adaptation, while Bio-C sealer exhibited the lowest microleakage. Therefore, no single sealer was superior across all evaluated parameters, highlighting the multifactorial nature of root canal sealer performance.

## Supplemental Information

10.7717/peerj.21621/supp-1 Supplemental Information 1 STROBE Checklist.

10.7717/peerj.21621/supp-2 Supplemental Information 2 Raw Data.

10.7717/peerj.21621/supp-3 Supplemental Information 3 Raw data of AH Plus Sealer used in Tables 2 and 3.

10.7717/peerj.21621/supp-4 Supplemental Information 4 Raw Data of Bio-c Sealer used for Tables 2 and 3.

10.7717/peerj.21621/supp-5 Supplemental Information 5 Raw data for Nanoseal used in Tables 2 and 3.

## Additional Information and Declarations

### Data Availability

The following information was supplied regarding data availability:

The data are available in the Supplemental files.
